# Dereplication of antimicrobial biosurfactants from marine bacteria using molecular networking

**DOI:** 10.1038/s41598-021-95788-9

**Published:** 2021-08-11

**Authors:** Albert D. Patiño, Manuela Montoya-Giraldo, Marynes Quintero, Lizbeth L. López-Parra, Lina M. Blandón, Javier Gómez-León

**Affiliations:** 1 Marine Bioprospecting Line, Marine and Coastal Research Institute “José Benito Vives de Andréis”-INVEMAR, Calle 25 No. 2-55, Playa Salguero, Santa Marta D.T.C.H., Santa Marta, Colombia; 2 Grupo de Investigación en Electroquímica y Medio Ambiente (GIEMA), Universidad Santiago de Cali, Calle 5 # 62-00, Santiago de Cali, Valle del Cauca, Colombia

**Keywords:** Metabolomics, Natural products

## Abstract

Biosurfactants are amphiphilic surface-active molecules of microbial origin principally produced by hydrocarbon-degrading bacteria; in addition to the bioremediation properties, they can also present antimicrobial activity. The present study highlights the chemical characterization and the antimicrobial activities of biosurfactants produced by deep-sea marine bacteria from the genera *Halomonas*, *Bacillus*, *Streptomyces*, and *Pseudomonas*. The biosurfactants were extracted and chemically characterized through Chromatography TLC, FT-IR, LC/ESI–MS/MS, and a metabolic analysis was done through molecular networking. Six biosurfactants were identified by dereplication tools from GNPS and some surfactin isoforms were identified by molecular networking. The half-maximal inhibitory concentration (IC_50_) of biosurfactant from *Halomonas* sp. INV PRT125 (7.27 mg L^−1^) and *Halomonas* sp. INV PRT124 (8.92 mg L^−1^) were most effective against the pathogenic yeast *Candida albicans* ATCC 10231. For Methicillin-resistant *Staphylococcus aureus* ATCC 43300, the biosurfactant from *Bacillus* sp. INV FIR48 was the most effective with IC_50_ values of 25.65 mg L^−1^ and 21.54 mg L^−1^ for *C. albicans*, without hemolytic effect (< 1%), and non-ecotoxic effect in brine shrimp larvae (*Artemia franciscana*), with values under 150 mg L^−1^, being a biosurfactant promising for further study. The extreme environments as deep-sea can be an important source for the isolation of new biosurfactants-producing microorganisms with environmental and pharmaceutical use.

## Introduction

The marine ecosystem is an important source of compounds with interesting biological activities that can be given economic or industrial value. In tropical countries like Colombia that has a great diversity of unexplored natural resources, especially the deep-sea habitats that could host a variety of microbial species have not yet been described^1^.

Deep-sea microorganisms in the Colombian Caribbean Sea have been little explored, and it is generally unknown what metabolites they can produce. It is possible to infer that, due to living in extreme conditions such as high pressure and salinity, shortages of light and nutrients, they have the ability to produce compounds that degrade complex carbon sources such as biosurfactants and chemical defense compounds like antimicrobials^2^.

Currently, microbial resistance is a serious health problem that affects humans^3^. It is estimated that 70% of pathogenic bacteria are resistant to at least one antibiotic. As consequence, the treatments are ineffective, and the infections are persistent. In addition, inappropriate behaviors like self-prescription facilitates the selection, persistence and dissemination of resistant microorganisms^4^. Therefore, it is necessary to search for new compounds with antimicrobial activity.

Once a bacterium is identified as a possible antimicrobial metabolite producer, the next step is the identification of the compound or groups of compounds responsible for the biological activity. The discovery and elucidation of the structure of these compounds involve several purification steps from the microbial culture. On the other hand, sometimes these metabolites are known because other microbes or organisms produce the same compounds or compounds with similar structural characteristics as in the case of the biosurfactants. In this sense, to facilitate this type of investigations, the data science focused on metabolites has emerged as metabolomics^5^.

Metabolomics is defined as the comprehensive analysis of metabolites in a biological specimen, combining high-throughput analytical chemistry and multivariate data analysis. It is based on experiments of chemical identification like nuclear magnetic resonance (NMR) and mass spectrometry (MS) analysis^6^. Metabolomics tools like molecular networking, are novel and agile for the identification of the compounds present in a mixture such as microbial culture media. In this sense, the information contributed by MS follows the design of molecular networking facilitating its identification as a procedure known like dereplication^7^.

The aims of this study were to identify the deep-sea bacteria as potential biosurfactant producers. To obtain extracts rich in biosurfactants, to characterize them chemically using thin-layer chromatography (TLC), infrared spectroscopy, chromatography coupled to mass spectrometry (HPLC–MS), and to know the relationship of the metabolic profile into each producer bacteria employing molecular networking. Besides, the antimicrobial activity of the biosurfactants was evaluated against human pathogens such as Methicillin-resistant *Staphylococcus aureus* subsp. *aureus* (MRSA) ATCC 43300 and *Candida albicans* ATCC 10231.

## Results

### Selection of biosurfactant producing bacteria: screening of oil-spread

**Figure 1:**
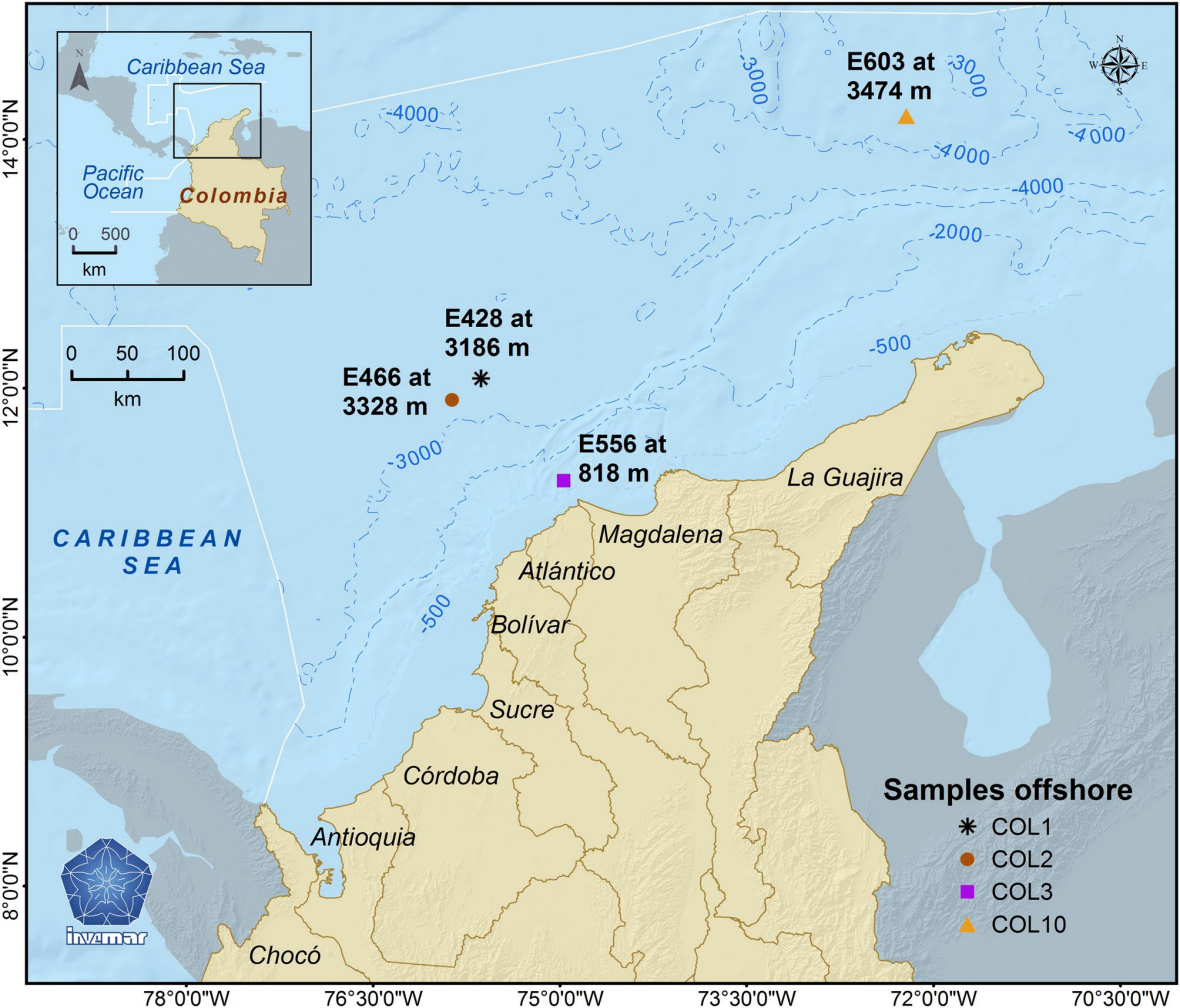
Exploration blocks in Colombian Caribbean Sea, denominated COL1 (12°4′51,30″N; 75°37′46,60″W), COL2 (11°54′28,40″N; 75°51′45,20″W), COL3 (11°15′28.54″N; 74°57′56.25″W) and COL10 (14°11′23.76″N; 72°13′2.81″W). " Map created in the Information Services Laboratory (LABSIS in Spanish) at Marine and Coastal Research Institute “José Benito Vives de Andréis”-INVEMAR, with the software ArcGIS 10.8".

Three hundred seventy-eight microorganisms were isolated from sediment samples taken from four different sectors of the Colombian Caribbean Deep-Sea (Fig. 1), they were of diverse depths: 818, 3186, 3328, and 3474 m. The strains were cultivated on Bushnell-Haas/Diesel fuel (DF) broth and sugar cane molasses before making the oil spread test. Finally, five bacteria were selected to continue with the molecular networking studies based on their morphological variety, and their capability to spread crude oil was the selection criteria (Table 1).

**Table 1:** Oil-spread results of five different deep-sea microorganisms, displacement of crude oil from biosurfactants produced in two carbon sources, diesel fuel (DF) and sugarcane molasses (SM).

No. catalogue MMNHC	Accesion code GenBank	Sampling station	Strain	OS in DF (mm)	OS in SM (mm)
INV PRT124	MK129413	E603 at 3474 m	*Halomonas* sp.	5.3 ± 0.4	6.0 ± 1.4
INV PRT125	MK129414	E603 at 3474 m	*Halomonas* sp.	3.0 ± 0.0	6.3 ± 0.6
INV FIR48	MK129309	E428 at 3186 m	*Bacillus* sp.	3.3 ± 0.4	5.3 ± 0.3
INV PRT82	MK129375	E466 at 3328 m	*Pseudomonas* sp.	5.0 ± 0.0	3.7 ± 0.6
INV ACT15	MK129407	E556 at 818 m	*Streptomyces* sp.	2.5 ± 0.7	2.0 ± 0.4

Media ± 1SD of three repetitions.

### Biosurfactant extraction performance and analyze by TLC

The five bacteria were cultured for seven days, and the biosurfactants (BS) were obtained from liquid ferment cell-free. The BS had an oily appearance and different colors (Figure S4); the production of BS obtained is showed in the Table 2.

**Table 2:** Amount of biosurfactants produced from *Halomonas* sp. INV PRT124, *Halomonas* sp. INV PRT125, *Bacillus* sp. INV FIR48, *Pseudomonas* sp. INV PRT82 and *Streptomyces* sp. INV ACT15.

No	No. catalogue MMNHC	Amount mg L^−1^
1	*Halomonas* sp. INV PRT124	12.3 ± 0.8
2	*Halomonas* sp. INV PRT125	42.0 ± 1.6
3	*Bacillus* sp. INV FIR48	96.5 ± 1.4
4	*Pseudomonas* sp. INV PRT82	45.1 ± 2.2
5	*Streptomyces* sp. INV ACT15	42.8 ± 3.2

Media ± 1SD of three repetitions.

The strain with the higher production of BS (96.5 mg L^−1^) was *Bacillus* sp. INV FIR48 this result is related to oil-spreading test that gets a halo of 5 mm, one of the highest of our tested strains (Table 1).

**Figure 2:**
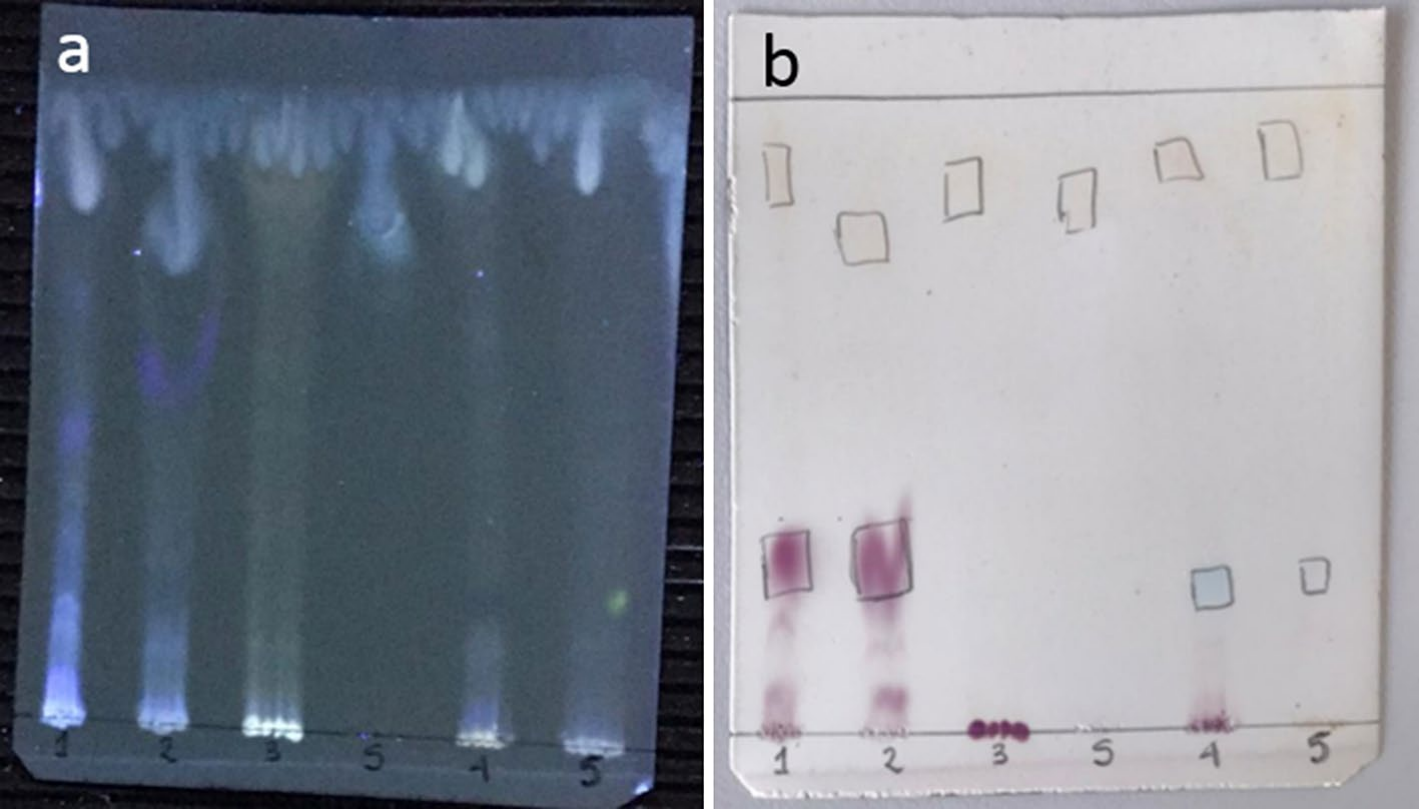
Chromatography thin layer of biosurfactant extracts (1) *Halomonas* sp. INV PRT124, (2) *Halomonas* sp. INV PRT125, (3) *Bacillus* sp. INV FIR48, surfactin (S), (4) *Pseudomonas* sp. INV PRT82 and (5) *Streptomyces* sp. INV ACT15. (**a**) Revealed UV-366 nm, (**b**) revealed with ninhydrin solution to 0.5% methanol*.*

The BS have different colors; this could hypothesize that each extract has a different metabolic composition. The analysis by TLC shows a preliminary chemist profile of the BS (Fig. 2a). The extracts were loaded on TLC, the numbers correspond to codes (Table 2) and correspond to surfactin. The TLC revealed with UVA 366 nm shows that all BS have different spots. Surfactin has a RF (Retardation Factor) = 0.84, the five BS have spots with RF in an interval of (0.78–0.89) (Fig. 2b).

### Biosurfactant characterization

#### FT-IR spectrum

**Figure 3:**
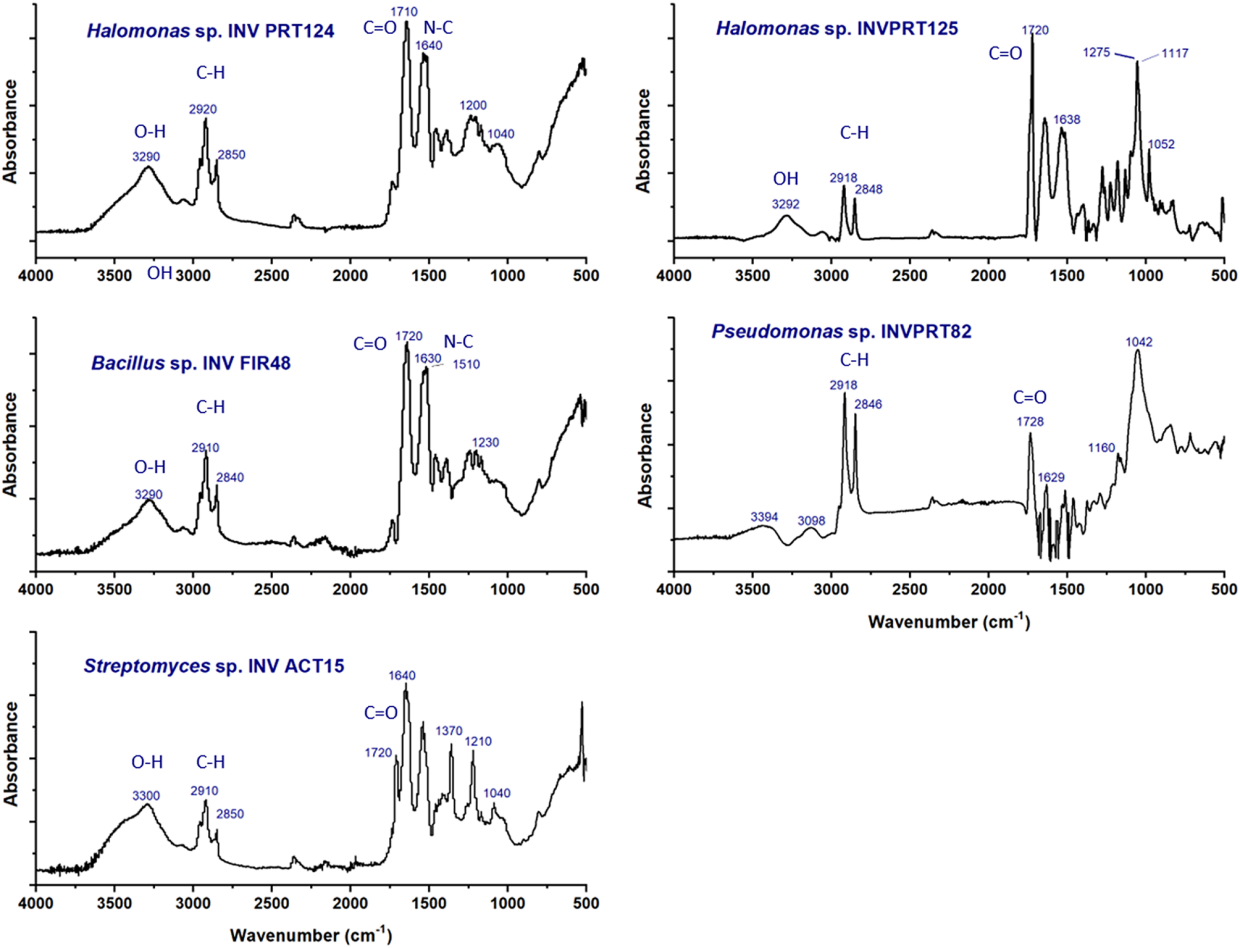
FT-IR spectra of biosurfactants extracts *Halomonas* sp. INV PRT124, *Halomonas* sp. INV PRT125, *Bacillus* sp. INV FIR48, *Pseudomonas* sp. INV PRT82 and *Streptomyces* sp. INV ACT15.

The spectra of biosurfactants (Fig. 3) show common bands. The bands observed at wavenumber 3394, 3292 and 3290 cm^−1^ were attributed to stretching OH group. The absorbance bands ranging from 2920 to 2840 cm^−1^ correspond to the stretching vibration of C–H. The absorbance peaks in 1710, 1720 and 1720 cm^−1^ were assigned to stretching C=O in esters groups, probably amides. The bands showed between 1640–1629 cm^−1^ were attributed to flexion N–C, this result confirms the presence of peptides. The absorption peaks, located at 1160, 1052, 1042 and 1040 cm^−1^ showed the presence of ester carbonyl groups (–CO bond) (Table S1).

#### Tentative identification of compounds by HPLC-MSMS

**Figure 4:**
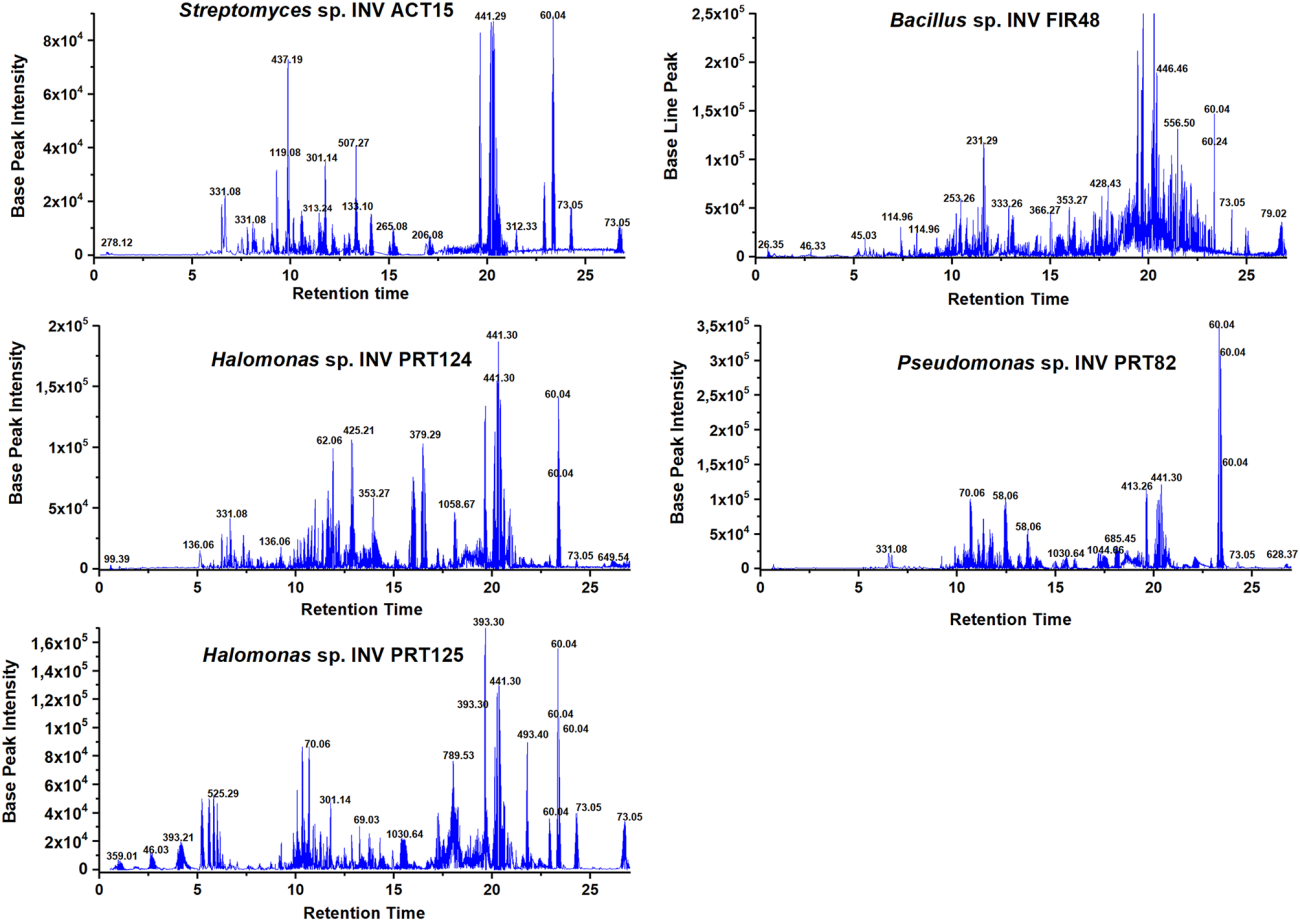
Chromatography profile UHPLC-MS/MS of biosurfactants extracts *Halomonas* sp. INV PRT124, *Halomonas* sp. INV PRT125, *Bacillus* sp. INV FIR48, *Pseudomonas* sp. INV PRT82 and *Streptomyces* sp. INV ACT15.

The BS were analyzed by liquid chromatography coupled to mass spectrometry; the total ion chromatograms (TICs) for each bacterium are shown in Fig. 4. In general, each species studied have a TIC with a huge number of peaks, the identification of compounds in mix in this case would not be successful due to the complexity of the TICs. It would be necessary to use a dereplication technique. The major number of peaks with higher intensity are between 10.0–12.0 min and 18.0–22.0 min. In the last interval, interesting peaks are detected with mass ionization [M + H]^+^ values of 1030.64 m/z, 1044.56 m/z, and 1058.67 m/z.

The raw data was processed in the software MZmine, the files were uploaded online to workflow GNPS: Global Natural Products Social Molecular Networking. GNPS is a mass spectrometry data base which facilitates the identification and discovery of compounds. The dereplication was done and the results are found in the next link https://gnps.ucsd.edu/ProteoSAFe/result.jsp?task=65fc168c2e01456d8a94a4ef086c4be0&view=group_by_compound. This would be the first proposal for a tentative identification of compounds existing in each biosurfactant, these results are shown in Table 3.

**Table 3:** Compounds indentified putatively in biosurfactants extracts from strains *Halomonas* sp. INV PRT124, *Halomonas* sp. INV PRT125, *Bacillus* sp. INV FIR48 and *Pseudomonas* sp. INV PRT82 by dereplication tool GNPS.

No	Compound	MZ error PPM	M/Z	INV FIR48	INV PRT82	INV PRT124	INV PRT125
1	Esperin	1	1036.69	✓	✓	✓	✓
2	[Leu7]surfactin C14i monomethyl ester	1	1036.69	✓			✓
3	Surfactin A C14	3	1022.68	✓	✓	✓	✓
4	Surfactin-D	3	1050.71	✓	✓	✓	
5	Plipastatin	6	731.39		✓		✓
6	[Val7]Surfactin C15ai	4	1022.68		✓	✓	✓

Thus, twenty-one compounds were identified with GNPS database, but the compounds that had a m/z error under to 6.0 ppm were considered in the putative identification^8^. The mass error compound represents the difference between observed experimental fragment m/z and theoretical fragment m/z reported in the database for each specific compound, so that parameter could be considered as the first criterion of identification also their MS/MS spectra was compared with the reported in the database (GNPS)^9^ (Figures S5–S10).

### Molecular networking analysis

The metabolic analysis of the five biosurfactants from bacteria was done through molecular networking with the data processed of UHPLC-MS/MS. Molecular networking was designed online in the platform GNPS and the results are available in https://gnps.ucsd.edu/ProteoSAFe/status.jsp?task=65fc168c2e01456d8a94a4ef086c4be0. The global molecular network (GMN) of the five BS is composed by 315 nodes, which were grouped into 25 clusters, it also showed that several clusters identify some compounds as 1-(9Z-Octadecenoyl)-sn-glycero-3-phosphocholine, 1-Stearoyl-2-hydroxy-sn-glycero-3-phosphoethanolamine esperin, plipastatin and surfactin.

**Figure 5:**
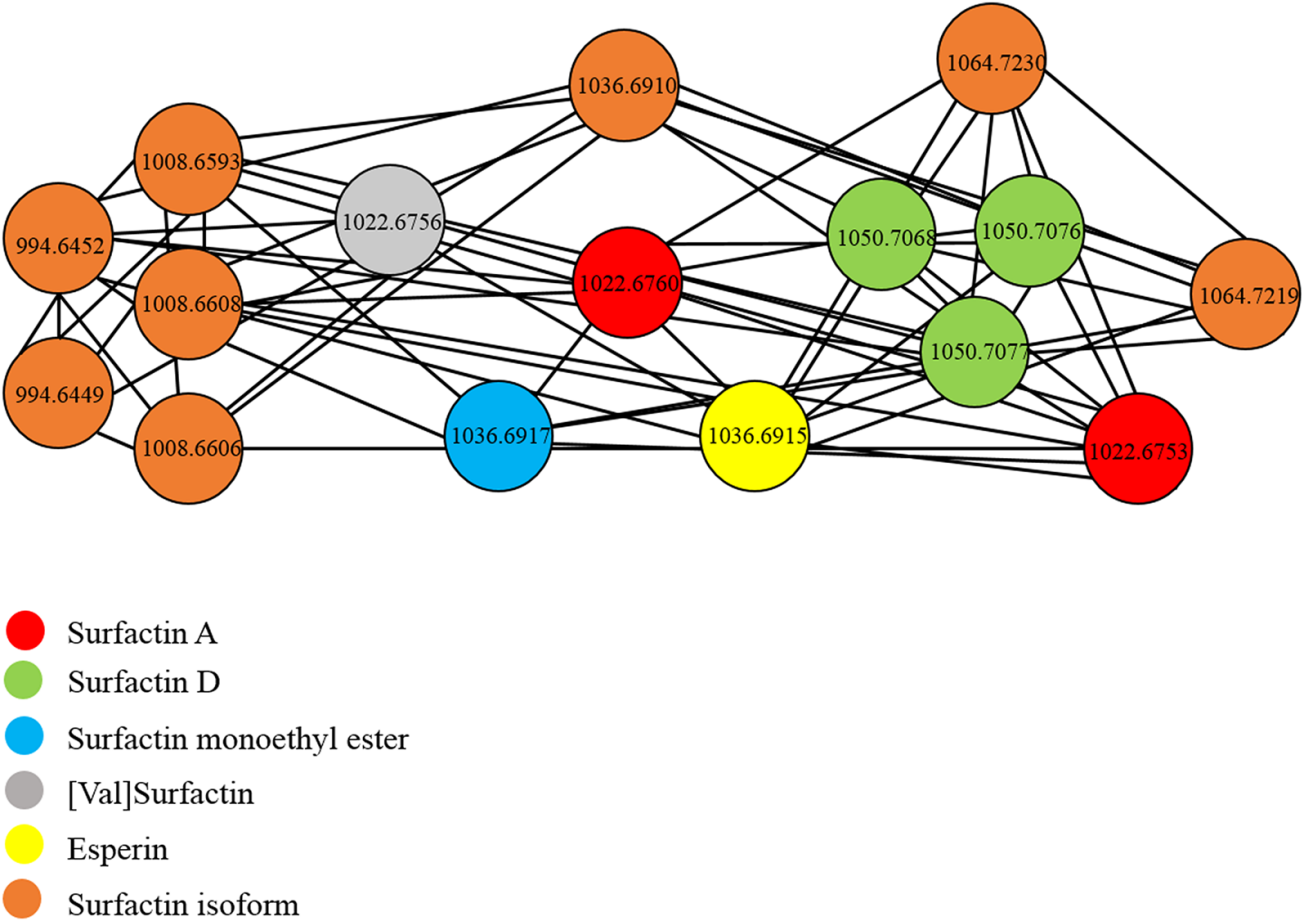
Molecular network of the molecular family of surfactins extracted from global molecular network of biosurfactants extracts *Halomonas* sp. INV PRT124, *Halomonas* sp. INV PRT125, *Bacillus* sp. INV FIR48 and *Pseudomonas* sp. INV PRT82.

The molecular network of the surfactin family (Fig. 5) has 16 nodes of different colors related to their molecular weight and identification of the compounds. The nodes are connected between them, the relation is because they have the same precursor ions and some similarity in the fragmentation. Thus, it is possible to relate and identify the different isoforms of surfactin. Those were identified by dereplication with GNPS spectra libraries. The nodes of orange color were not possible to identify by GNPS. The value of molecular weight and connection with other surfactin isoforms infer that are isoforms too.

### Antimicrobial activity

The BS presented antimicrobial activity against both pathogens with MIC values from 187.5 to 500 mg L^−1^ onwards. *Bacillus* sp. INV FIR48 has the higher activity against MRSA, with a MIC value of 500 mg L^−1^ that inhibited 83.7% of the pathogen. *C. albicans* ATCC 10231 was inhibited in 90.2% by the BS from *Halomonas* sp. INV PRT125, with a MIC value of more than 187.5 mg L^−1^, followed by *Halomonas* sp. INV PRT124 with a MIC value of more than 375 mg L^−1^, concentration that inhibited 90.3% of the yeast. *Bacillus* sp. INV FIR48 (MIC value of more than 375 mg L^−1^) and *Streptomyces* sp. INV ACT15 (MIC value of more than 500 mg L^−1^) inhibited *C. albicans* in 89%. *Pseudomonas* sp. INV PRT82 showed inhibition of less than 20% at the higher concentration evaluated of 500 mg L^−1^, like the results obtained against MRSA for BS from *Halomonas* sp. INV PRT124 and *Streptomyces* sp. INV ACT15.

Therefore, the half-maximal inhibitory concentration (IC_50_) of BS is shown in Table 4. The inhibition values against *C. albicans* exhibit that the most effective BS was from *Halomonas* sp. INV PRT125 which revealed an IC_50_ of 7.27 mg L^−1^. Also, *Halomonas* sp. INV PRT124 extract express an IC_50_ of 8.92 mg L^−1^. These IC_50_ values were around eight times higher than the positive control Nystatin that showed an IC_50_ of 0.36 mg L^−1^. For Methicillin-resistant *S. aureus* ATCC 43300 (MRSA) the BS *Bacillus* sp. INV FIR48 revealed an IC_50_ of 25.65 mg L^−1^ and the positive control was Rifampicin (IC_50_ of 0.01 mg L^−1^). According to results and knowing that for compounds present in the mixture the IC_50_ value should be below 100 mg L^−1^, all the BS of this study are promissory for the isolation and chemical characterization, with the objective of using them as formulation for an antiseptic agent or for pharmaceutical uses.

**Table 4:** BSE values of Half-maximal inhibitory concentration (IC_50_) for strains *Halomonas *sp. INV PRT124, *Halomonas* sp. INV PRT125, *Bacillus* sp. INV FIR48, *Pseudomonas* sp. INV PRT82 and *Streptomyces* sp. INV ACT15.

No. catalogue MMNHC strains	IC_50_ MRSA ATCC 43300 (mg L^−1^)	IC_50_ *C. albicans* ATCC 10231 (mg L^−1^)
*Halomonas* sp. INV PRT124	> 500	8.92
*Halomonas* sp. INV PRT125	93.48	7.27
*Bacillus* sp. INV FIR48	25.65	21.54
*Streptomyces* sp. INV ACT15	> 500	20.94
*Antibiotics*		
Rifampicin	0.01	N/A
Nystatin	N/A	0.36

*N/A* not apply.

### Toxicity of biosurfactants

The toxicity that BS presented over the aquatic model organism, brine shrimp larvae *Artemia franciscana* was moderate under 300 mg L^−1^, only *Halomonas* sp. INV PRT124 showed high toxicity at 500 mg L^−1^ (52.5%). In addition, none of the BS have hemolytic effect over human cells when evaluated in 2 concentrations (333 mg L^−1^ y 166 mg L^−1^), with percentages lower than 1% which lead us to think that the quantity of the active compounds is lower than the sensibility of the hemolytic assay^10^.

## Discussion

The Caribbean Sea is a semi-enclosed basin of the western Atlantic Ocean with an average depth of 2400 m being a 75% deeper than 1800 m approximately^11^. Worldwide, significant efforts have been made to cultivate marine microorganisms from deep-sea environments under controlled laboratory conditions obtaining a low representation of these. Nevertheless, the few representatives available are highly valuable for discovering information about unusual types of metabolism^12^. In marine ecosystems, the bacterial production of amphiphilic surface active molecules like biosurfactants can be involved in the microbial competition (antimicrobial properties), nutrition (favoring the accession to water—insoluble substrates) and survival^13^.

Bacterial growth was noted in the presence of hydrocarbon (diesel fuel) as the only carbon source, which suggests the possibility of the production of biosurfactants or other metabolites that help with the digestion of the mentioned carbon source^14^. This feature makes them candidates for biodegrading contaminated sources, being this an emerging technology^15^. Exploring these biological processes for petroleum biotechnology and other potential applications of these biomolecules due to their biodegradability, allowed them to be used in the cosmetic and pharmaceutical fields^16^.

TLC analysis shows RF values close to surfactin that could indicate its presence or of analogues. Lane 1 and 2 have a similar profile, probably because strains belong to the same genus (*Halomonas*) and produce similar metabolites^17^. The purple and yellow spots correspond to the reaction of amino acids (AA) with ninhydrin. Purple corresponds to non-polar AA like glycine, alanine, valine, leucine. The yellow spots correspond to polar or negatively charged AA such as proline, asparagine, glutamine. AA are present in surfactin and iturin structure^18^.

The set of bands suggest the presence of the lipopeptides in all extracts^19^. There is a high correlation between *Halomonas* sp. INV PRT124 and *Bacillus* sp. INV FIR48 in their IR profiles. However, for *Halomonas* sp. INV PRT125 there are signals close to 1117 cm^−1^ corresponding to stretching C–O–C bonds of structures with lipopeptide character, and for *Pseudomonas* sp. INV PRT82 there is a strong signal found at 1042 cm^−1^ involving probably ester linkages of surfactin-C14 and surfactin-C15^20^.

The analysis of the TICs of BS allowed to identify which peaks were common between bacteria, the most intense and selecting the interesting peaks to analyze their MS^2^ fragmentation or also analyzing the MS^2^ data with a software of data processing. The peaks with m/z value of 1030.64 m/z, 1044.56 m/z corresponds to characteristic peaks of surfactin families^21^ and the peak 1058.67 m/z corresponds to iturin (lipopeptide)^22^. The other compounds were identified although data was processed in MZmine and using database from GNPS (Table 3). It was possible to find common compounds in all strains, these belong to surfactin family and were identified in the strains INV FIR48, INV PRT82, INV PRT124 and INV PRT125, also it was identified that plipastatin compound belonged to iturin family. Surfactin and its isoforms are produced by the genera *Bacillus*, *Pseudomonas*, and *Halomonas*, this result could explain the biosurfactant activity in oil-spread assay using carbon source SM^23^, where the strains of *Streptomyces* did not produce surfactin, explaining the low activity in the same assay.

The strains studied produce different surfactin isoforms, but the strains **INV PRT82** (*Pseudomonas* sp.) and **INV PRT125** (*Halomonas* sp.) produce the major number of isoforms. This result could be interesting in environmental studies of bioremediation. The metabolomic study of the surfactin isoforms brings results to the diversity of metabolic of marine bacteria producer of biosurfactants.

The unknown surfactin isoforms were identified through their molecular weight, mass spectra report in literature and connections of the nodes in the molecular network (Table 5). For example, the isoform 1064.723 m/z has a mass spectrum with characteristic peaks of surfactin (fatty acid chains). The analysis of MS^2^ (Figure S17) shows a loss weight of 113.0 that corresponds to leucine, a loss of 133.0 assigned to aspartate, a loss of 117.0 belonging to valine, other loss of 113.0 that also corresponds to leucine. These fragments are characteristic of surfactin A. The node of this isoform has a connection with surfactin A and its molecular weight match with surfactin A C17^23,24^. Thus, the unknown surfactin isoforms were identified putatively in that way.

**Table 5:** Surfactin isoforms identified through molecular networking in deep-sea marine bacteria *Halomonas* sp. INV PRT124, *Halomonas* sp. INV PRT125, *Bacillus* sp. INV FIR48 and *Pseudomonas* sp. INV PRT82.

Structure	Molecular formula	[M + H]^+^	INV FIR48	INV PRT82	INV PRT124	INV PRT125
[Val7]Surfactin C13	C_50_H_87_N_7_O_13_	994.6452	✓			✓
Surfactin C12	C_50_H_87_N_7_O_13_	994.6449	✓			✓
Surfactin A C13	C_51_H_89_N_7_O_13_	1008.6593		✓		✓
Surfactin C15	C_51_H_89_N_7_O_13_	1008.6608	✓		✓	✓
Surfactin A C13	C_51_H_89_N_7_O_13_	1008.6606		✓		✓
Surfactin B C16	C_53_H_93_N_7_O_13_	1036.6910		✓		✓
Surfactin A C17	C_55_H_97_N_7_O_13_	1064.7230	✓	✓		✓
Surfactin monoethyl ester C16	C_55_H_97_N_7_O_13_	1064.7219		✓		✓

These results support the production of secondary metabolites with antimicrobial activity. According to the metabolic analysis, *Halomonas* sp. INV PRT125 produces a wide range of biosurfactants like surfactin A C14, surfactin-D, [Leu7]surfactin monoethyl ester C14, [Val7] surfactin C15 and esperin. Similarly, *Halomonas* sp. INV PRT124, *Pseudomonas* sp. INV PRT82 and *Bacillus* sp. INV FIR48 produced surfactin isoforms in common. It should be because most of the reports on surfactin obtention refer to genus *Bacillus* as its main producer^25^. However, there are few studies describing the production of lipopeptide families by Gram-negative bacteria and only production has been presented in bacteria isolated from soil samples of the genera *Enterobacter*, *Citrobacter* and *Pseudomonas*^26,27^. Therefore, the surfactin isoforms production by the genera *Halomonas* and *Pseudomonas* are interesting for the discovery of other producing strains, especially for isolation of marine biosurfactants, which have not been widely explored^28^. In this sense, the extreme environments as deep-sea, can be an important source for the isolation of new biosurfactant-producing microorganisms.

The antimicrobial activity of Surfactin A C14 obtained from marine species of genus *Bacillus* and evaluated against *C. albicans* has been studied^1^ reporting an anti-fungal MIC values of C14-surfactin of more than 100 mg L^−1^. In our study, the BS from *Bacillus* sp. INV FIR48 exhibited IC_50_ values of 21.54 mg L^−1^ (MIC of more than 375 mg L^−1^), this effective concentration (below 100 mg L^−1^) shows the antimicrobial potential of deep-sea bacteria. Additionally, these observations of MIC values, may have proximity to that found in BS of *Halomonas* sp. INV PRT124 and *Halomonas* sp. INV PRT125 against the yeast *C. albicans* ATCC 10231. It is known the surfactin to interact with the cell membrane and disturb the membrane’s stability^29^, hence the novelty production of surfactin A C14 by species of genus *Halomonas* is promising for further exploration of other biological activities.

On the other hand, *Bacillus* sp. INV FIR48 and *Halomonas* sp. INV PRT125 showed antimicrobial activity against MRSA. It is interesting for the study that strains belonging to different bacterial genera produced surfactin isoforms in common, like Surfactin A C14, Surfactin D and [Leu7] surfactin monoethyl ester C14. Liu reports the ability of surfactin produced by *Bacillus subtilis* for affecting *S. aureus* biofilm formation, also the lipopeptide attacks the quorum sensing (QS) system in the Gram-positive bacteria *S. aureus*^30^. In general, the Gram-positive bacteria are considered more sensitive to natural compounds due to the structure of their cell walls, the lipopeptides pore formation in membranes, leading to an imbalance in transmembrane ion fluxes and cell death^31^.

The BS toxicity was evaluated using *Artemia franciscana* and a hemolytic assay. Brine shrimp, *Artemia* spp., is an invertebrate zooplankton involved in the energy flow of the food chain in many of saltwater lake ecosystems. Due to its high offspring production and ease of culture, short life cycle, ready availability, and small body size, this aquatic invertebrate is widely used in toxicity assays^32^. On the other hand, the term hemolysis refers to the phenomenon of rupture or lysis of the erythrocyte membrane due to the action of physical or chemical agents causing the release of hemoglobin. In the body, hemolysis causes oxygen deficiency in the tissues, release of reticulocytes into the bloodstream and physical wear and tear of the bone marrow. The hemolysis test is very useful when it comes to obtaining possible compounds or drug candidates, since in many cases blood is one of the transport frontiers of these molecules^33^.

Studies showed that surfactin isoforms, like C14 and C15, from *Bacillus subtilis* are thought to be related to antimicrobial activity^34^. Also, the authors notice that hemolytic activity increased within higher hydrophobicity of the compound, but other investigations^35^ revealed the opposite, that the hemolytic activity of synthetic surfactin analogues is not related to their hydrophobicity.

Lastly, in our study was found that the deep-sea bacteria produced more than one biosurfactant and it is necessary the purification to confirm that antimicrobial activity is due to surfactin. Furthermore, the novelty of production of surfactin isoforms by genus *Halomonas* and *Pseudomonas* is promising as new source of marine biosurfactants to continue exploring other possible specific biological activities. The activity against Methicillin-resistant *S. aureus* and *C. albicans*, non-ecotoxicity against *Artemia franciscana* neither toxicity over human red blood cells, shows promising results for further investigation.

## Materials and methods

### Culture medium

Marine agar (Difco), Bushnell-Haas broth modified (magnesium sulphate (0.2%), calcium chloride (0.02%), monopotassium phosphate (1.0%), dipotassium phosphate (1.0%), sodium nitrate (1.0%), ferric chloride (0.05%)) and supplemented with Sugarcane molasses (SM) (1.0%), TSA and Muller-Hinton broth.

### Bacteria isolation, culture, and molecular identification

Deep-sea bacteria were isolated from sediments samples obtained from the exploration blocks in Colombian Caribbean Sea, denominated as COL1 (12°4′51,30″N; 75°37′46,60″W), COL2 (11°54′28,40″N; 75°51′45,20″W), COL3 (11°15′28.54″N; 74°57′56.25″W) and COL10 (14°11′23.76″N; 72°13′2.81″W), in the sampling stations E466 at 3328 m, E428 at 3186 m, E556 at 818 m and E603 at 3474 m, respectively. All of the 378 bacterial strains isolated from these stations were screened to notice their biosurfactant production^36^. For molecular identification, the 16S rRNA gene was amplified using the following prokaryotic universal primers: F (5′-AGA GTT TGA TCC TGG CTG AG-3′) and R (5′-GGT TAC CTT GTT ACG ACT T-3′)^37,38^. The 16S RNA gene was sequenced in CNSG and Corpogen-Colombia. The identity of the sequences was compared with reference taxonomies hosted by SILVA (RDP, Greengenes, and SILVA)^39–41^ using the least common ancestor (LCA) method and searching five of the nearest sequences with a similarity superior at 97% (Figures S1, S2 and S3) and sequences were deposited in GenBank (Table 1). Therefore, *Halomonas* sp. INV PTR124, *Halomonas* sp. INV PTR125, *Bacillus* sp. INV FIR48, *Pseudomonas* sp. INV PRT82 and *Streptomyces* sp. INV ACT15 were the strains used in this study for biosurfactant producing bacteria. The set of bacteria was deposited at the Marine Museum of Natural History of Colombia–Makuriwa (MMNHC).

Bushnell-Haas broth modified with 1% sodium chloride and 1% Diesel fuel (DF) as carbon source were used as production medium for the culture of microorganisms. One or two colonies were suspended in 10 mL of medium until 0.5 McFarland scale, aliquots of 1 mL were used for inoculated 25 mL of production medium. After 7 days of incubation at 30 °C and 140 rpm, 1 mL of the cell-free culture was obtained by centrifugation at 9520 g, for 10 min at room temperature.

### Oil-spreading (OS) test of biosurfactants

In a petri dish containing 40 mL of distilled water, 20 µL of crude oil were poured in a thin layer on the surface. Then 20 µL of cell-free culture were added carefully in the center of the layer, the diameter of spreading was measured in mm. As negative control, 20 µL of the broth without inoculum was used, and 20 µL of neutral detergent (Extran MA 02) as positive control of displacement, each one in different petri dishes. The assay was done by triplicate.

A positive result was the spreading of the crude oil and a zone of emulsification on the surface^42^. Microorganisms that had positive results were cultured in Bushnell-Haas broth modified now with 1% sugarcane molasses (SM), to evaluate the production of metabolites in different carbon sources like this agro-industrial waste.

### Extraction of biosurfactants

The cells were removed by centrifugation at 2705 g for 20 min at 4 °C. The pH was adjusted to 2.0 with 6 M HCl, and the supernatant was incubated overnight at 4 °C to improve the precipitation. The precipitate was then collected by centrifugation at 2705 g for 30 min at 4 °C. Those were washed with HCl 0.1 M and suspended in water, its pH was adjusted 8.0, this suspension was freeze-dried. Finally, the BS were extracted with a mix chloroform: methanol (65:15) and evaporated under reduced pressure in a rotary evaporator at 40 °C^24^. The BS were weighted, and the production was calculated.

### Characterization of biosurfactants

#### Chromatography TLC plates analysis

The biosurfactants and surfactin were dissolved in a chloroform: methanol (13:3) mixture and subjected to thin layer chromatography (TLC) Silica Gel 60 F254, this was carried out in the mobile phase of the same mixture. The spots were revealed under UVA radiation (366 nm). Then the plates were immersed in a solution to 0.5% of ninhydrin/methanol for 10 s and heated to 100 °C for 1 h^43^.

#### Spectroscopy FT-IR analysis

The spectrums of attenuated total reflectance (ATR) were obtained using a FTIR spectrophotometer IR Tracer-100 (Shimadzu), with a detector DLATS and a horizontal reflection accessory ATR (ATR-MIRacle PIKE). Each spectrum was run with an average of 64 scans and with a resolution of 8 cm^−1^^44^. The signal assignment was performed considering the table of infrared spectroscopy of Sigma Aldrich^45^.

#### Liquid chromatography-MS/MS (LC–MS/MS) analyses

LC–MS/MS was performed in a UHPLC Thermo Dionex Ultimate 3000 coupled with HRMS (Impact II, Bruker Daltonics Corporation, USA) equipped with an electrospray ionization source (ESI). The chromatography system used an analytic column C18 (LC column Kinetex 1.7 µm particle size, 100 × 2.1 mm). The mobile phase is a binary solvent system consisting of A (water, 0.1% FA) and B (ACN, 0.1% FA). The elution conditions were “discontinuous” gradient as follows: 10% B for 5 min, 10–70% B over 7 min, 70% B for 5 min, 70–99% B over 4 min and 99% B for 6 min. The injection volume was 1 µL, the flow rate of 0.4 mL/min. Ionizations were acquired in positive ion mode using electrospray ionization mass spectrometry (ESI–MS) with capillary temperature, and the voltage was set at 200 °C, 3.5 kV, respectively. The scan ranged from m/z 50 to 1200. The obtained data were processed by Compass DataAnalysis software (Bruker)^46^.

### MS/MS data processing to molecular networking

Raw data from UHPLC-MS/MS analysis were converted to. mzXML format with Bruker’s Compass DataAnalysis software. LC–MS data was then preprocessed with the open-source software MZmine and consisted of peak detection, isotopes removal, peak matching, and peak filling. Peak detection was performed in three steps: (1st) mass detection with noise value = 500; (2nd) chromatogram builder with minimum time span = 0.01 min, minimum height = 3000 and m/z tolerance = 5.0 ppm; (= 3rd) deconvolution with peak width = 0.01–0.5 min, noise = 5000. Isotopes were removed using the isotopic peak grouper with m/z tolerance = 5.0 ppm, retention time tolerance = 0.1 min and minimum standard intensity = 5000. Then, a filter was applied to keep only those ions with at least 2 peaks in their isotope pattern. Peak matching among samples was performed using the join aligner with m/z tolerance = 5.0 ppm^47^. The final step was to export two files in format .mgf and .CSV, both files were uploaded to the global natural product social (GNPS) molecular networking tools^48^.

Finally, the maximum size of a molecular family was set to 100, and the lowest scoring edges were removed from molecular families until the molecular family size was below this threshold. The spectra in the network were then searched against GNPS spectral libraries^48,49^.The molecular networks were visualized using software Cytoscape version 3.7.2.

### Antimicrobial assays

#### Broth microdilution test

Antimicrobial activity evaluation of BS was performed according to the methodology described by Quintero^50^. Biosurfactants were suspended in 2% DMSO and concentration values from 6.25 to 500 µg mL^−1^ were evaluated through broth microdilution test as recommended by CLSI^51^. It was carried out in 96-well microplate (Corning F-bottom) by triplicate, against two pathogenic microorganisms: Gram-positive, round-shape bacteria Methicillin-resistant *Staphylococcus aureus* subsp. *aureus* ATCC 43300 (MRSA), and the opportunistic pathogenic yeast *Candida albicans* ATCC 10231. Each pathogenic strain in exponential growth phase was suspended in 10 mL of sterile Mueller–Hinton broth until obtaining 0.5 McFarland scale concentrations (10^8^ CFU mL^−1^), then they were exposed to the BS. Same suspension of pathogens was used as grown control and antibiotics (Rifampicin and Nystatin) were used as inhibition control. The plates were incubated at 30 °C for 24 h and the optical density (OD) was measured at 600 nm in the Multiskan GO spectrophotometer (Thermo Scientific). The inhibition and growth percentage were obtained by the following equations:

1 $$\% Growth = \frac{{\left( {OD_{Treatment} - OD_{Blank} } \right)}}{{OD_{Positive\;control} }} \times 100$$

2 $$\% Inhibition = 100 - \% Growth$$

The half- maximal inhibitory concentration (IC_50_) was calculated in accordance with the Clinical laboratory Standards Institute (CLSI) standard procedure for in vitro antimicrobial susceptibility testing^52^.

### Toxicology assays

#### Short-term lethal toxicity test with brine shrimp larvae

Ecotoxicity of the BS were measured adjusting the method of brine shrimp lethality test by Rajabi^53^, for Brine shrimp larvae. Dried cysts were cultured 48 h before in artificial sea water 35 ppt at room temperature (25–30 °C) under aeration and continuous illumination (2000 LUX), only the larvae (nauplii) hatched were selected for the assay.

The BS ecotoxicity was evaluated within concentration values from 150 to 500 (mg L^−1^) in 96-well microplates. BS were solved in 1.5% Methanol. Each well containing 10 nauplii in 200µL of artificial sea water and the correspondent concentrations were poured by quadruplicate, water was used as negative control, copper sulfate (CuSO_4_·5H_2_O) as positive control and 1.5% Methanol as solvent control. The plate was incubated for 24 h under illumination, the surviving organisms were counted for each well under a stereomicroscope (Carl Zeiss STeREO Discovery V.8). Lethality percentage was calculated and ecotoxicity was determined with this scale: 0–10% Non-toxic, 11–50% lowly toxic, 51–90% highly toxic and 100% extremely toxic^54^.

#### Hemolysis assay

This study was made according to resolution No. 008430 of 1993 of the Ministry of Health of Colombia^55^. All methods were performed in accordance with the ethical guidelines and regulations, and informed consent was obtained from the participating individual. Primary culture of human cells was obtained from blood of a healthy volunteer, according to^56^ modified method. The erythrocytes were collected from peripheral human blood (A positive) in vacutainer K_2_EDTA tube (BD) by centrifugation at 2705 g for 5 min at room temperature (25–30 °C), then 3 washes were made with 1X PBS rejecting the supernatant (or blood plasma) in each. At last, the hematocrit (pellet) was solved in 1X PBS (1:10) which means a suspension of 5 × 10^8^ g mL^−1^. 300 µL of the hematocrit solution were poured in a sterile microtube and 300 µL of the BS (solved in DMSO previously). The tubes were incubated for 1 h at 37 °C, then the tubes were centrifuged at 1350 g for 10 min, and 100 µL were poured in each well of the 96-well plate (by triplicate). Absorbance was measured at 541 nm in a Thermo Scientific Multiskan GO Microplate Spectrophotometer. The hematocrit solution without treatment was used as negative control, hematocrit solved in 1X PBS (1:10) and hematocrit in 1% DMSO. Triton X-100 and deionized (purified) water were used as positive control. The toxicity was calculated as the relative percentage over the positive control (Triton X-100) and values < 10% were accepted^33^.

## Supplementary Information

Supplementary Information.
