# Supplementary material for: Dereplication of antimicrobial biosurfactants from marine bacteria using molecular networking
Source: Sci Rep. 2021 Aug 11;11:16286. doi: 10.1038/s41598-021-95788-9 (PMC8357792; doi:10.1038/s41598-021-95788-9)
Supplement: Supplementary file 1 — Supplementary Information. [file 41598_2021_95788_MOESM1_ESM.docx]

Supporting information: Dereplication of antimicrobial biosurfactants from marine bacteria using molecular networking

Albert D. Patiño^1^, Manuela Montoya-Giraldo^1^, Marynes Quintero^1^, Lizbeth L. López-Parra^2^, Lina M. Blandón^1*^, Javier Gómez-León^1^

**^1^Laboratory of Marine Bioprospecting, Marine and Coastal Research Institute “José Benito Vives de Andréis”-INVEMAR, Calle 25 No. 2‑55, Playa Salguero, Santa Marta D.T.C.H., Santa Marta, Colombia**

**^2^Grupo de Investigación en Electroquímica y Medio Ambiente (GIEMA), Universidad Santiago de Cali, Calle 5 # 62-00, Santiago de Cali, Valle del Cauca, Colombia**

*Corresponding author: Dra. Lina Marcela Blandón

Marine and Coastal Research Institute “José Benito Vives de Andréis”- INVEMAR,

Calle 25 No. 2-55, Playa Salguero, Santa Marta D.T.C.H., Colombia.

E-mail: lina.blandon@invemar.org.co, lmblando@unal.edu.co

Phone: +575 4328600 ext 156


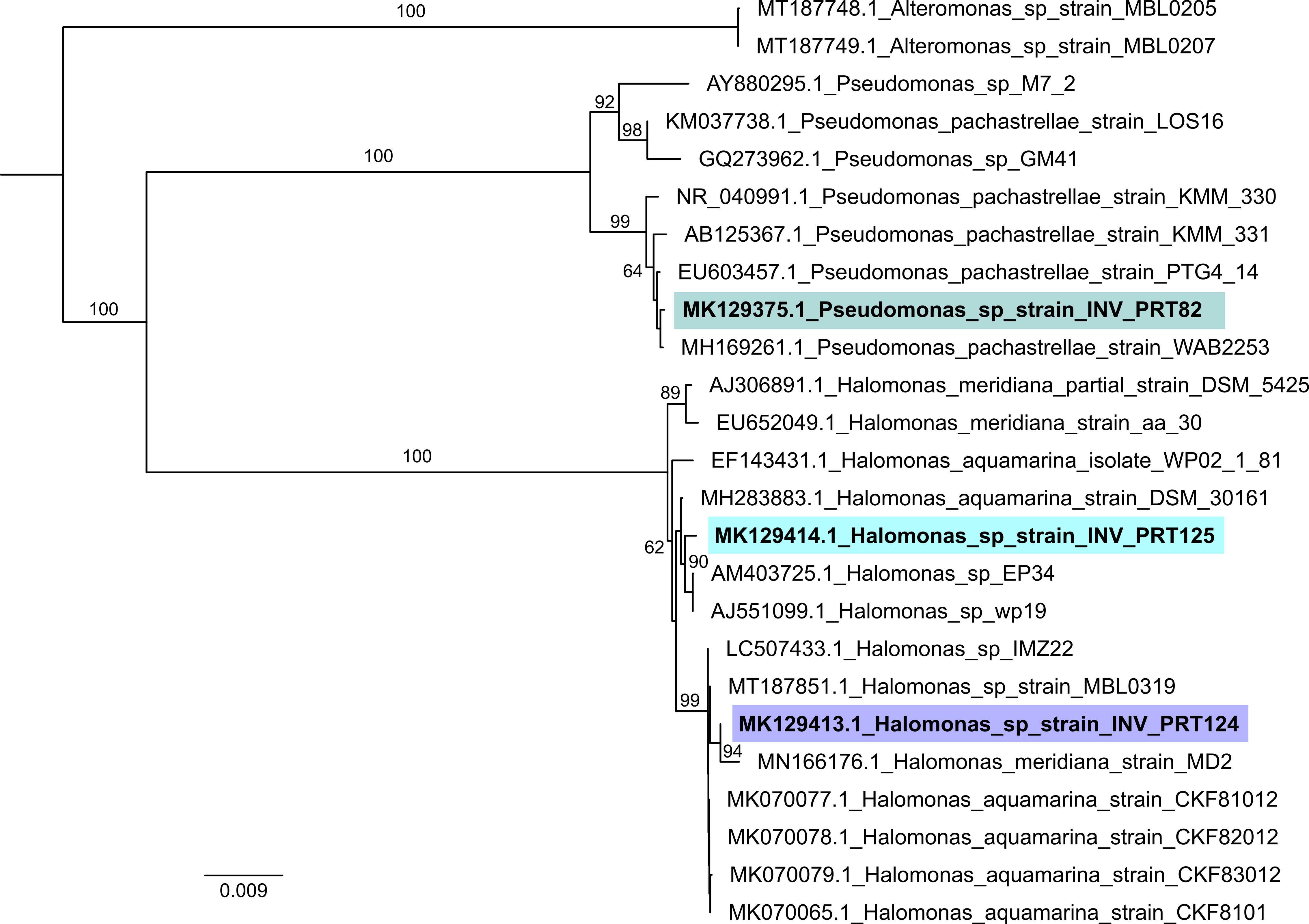


**Figure S1.** Phylogenetical tree of strains Halomonas sp. INV PRT124, Pseudomonas sp. INV PRT182 and Halomonas sp. INV PRT125.


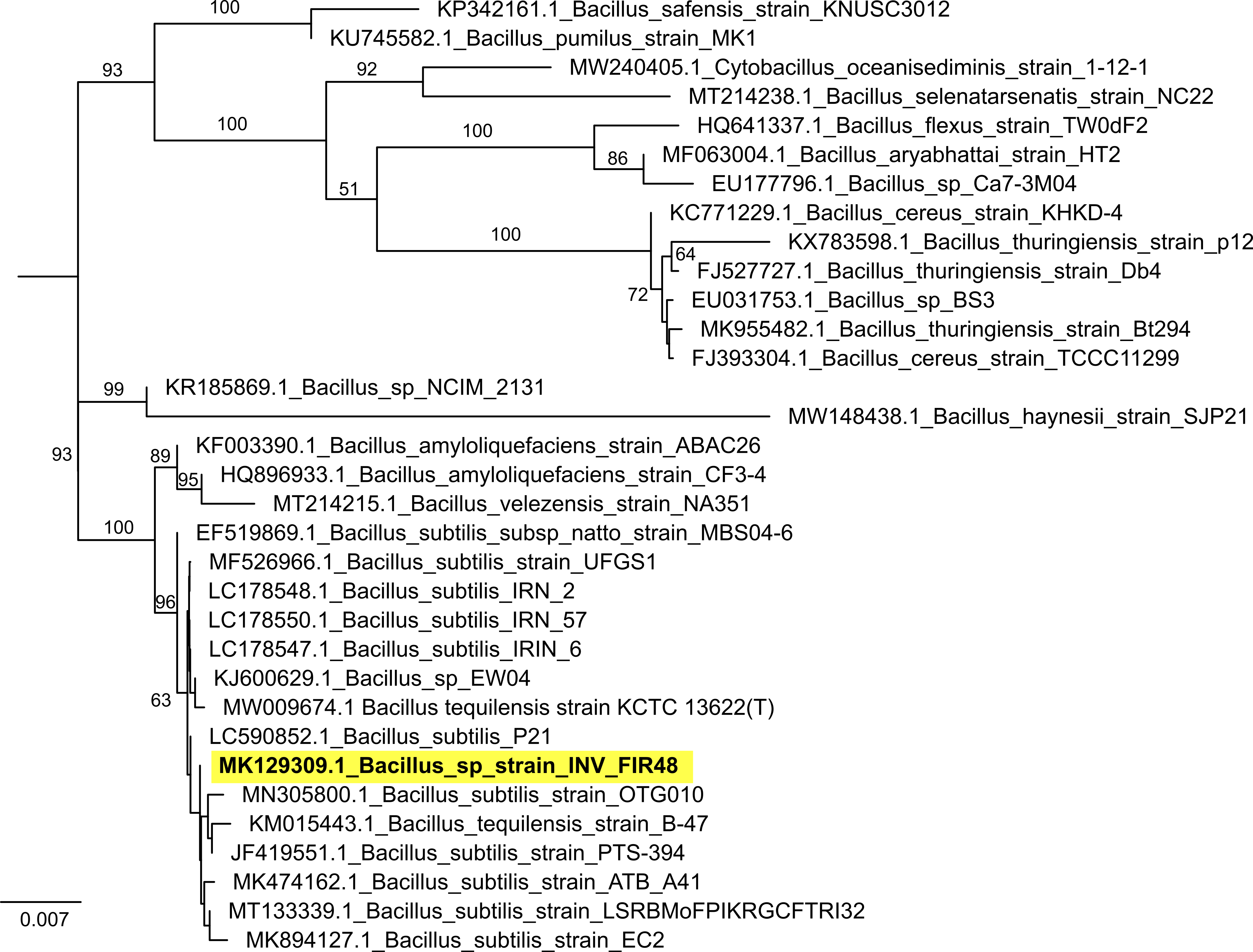


**Figure S2.** Phylogenetical tree of strain Bacillus sp. INV FIR48


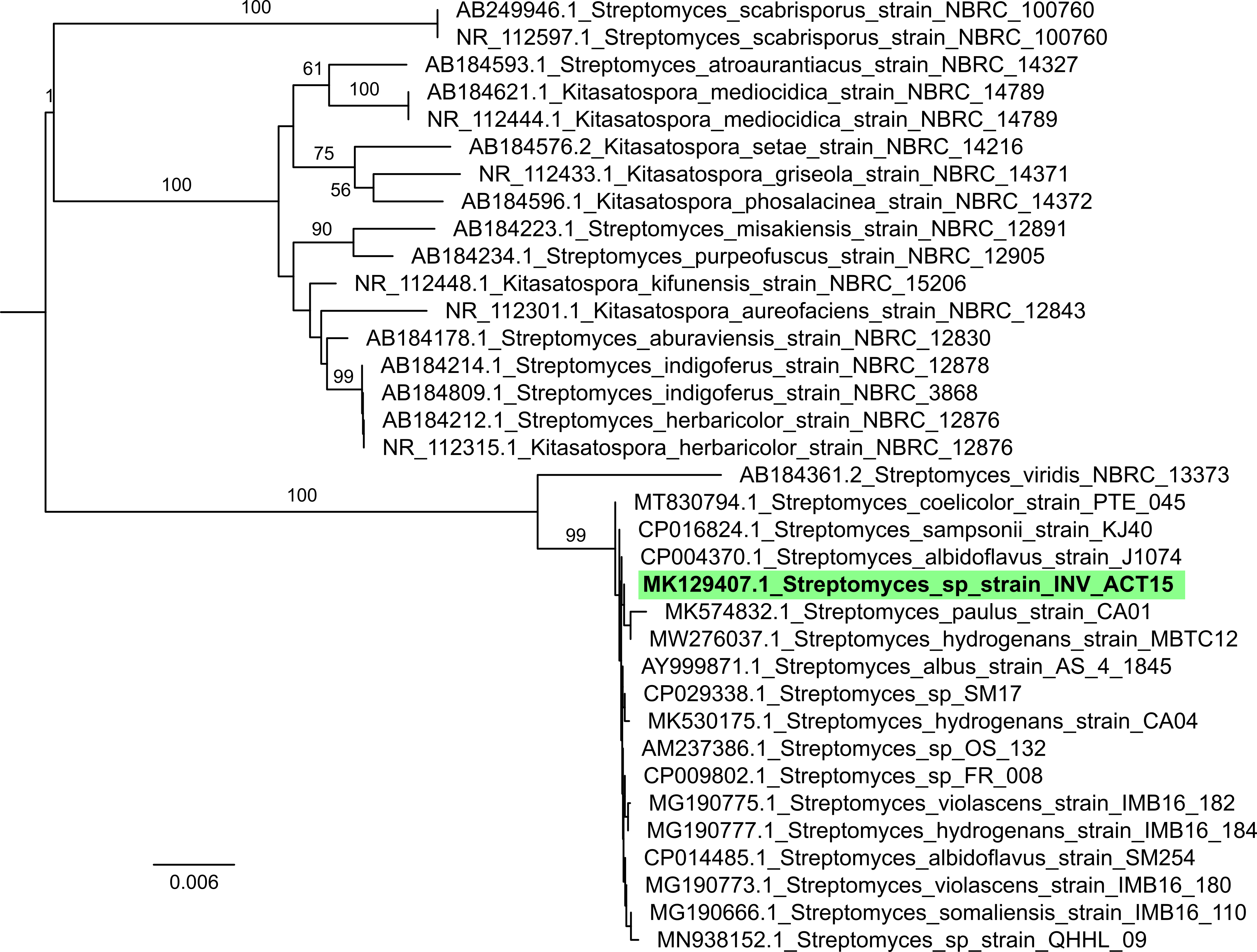


**Figure S3.** Phylogenetical tree of strain Streptomyces sp. INV ACT15


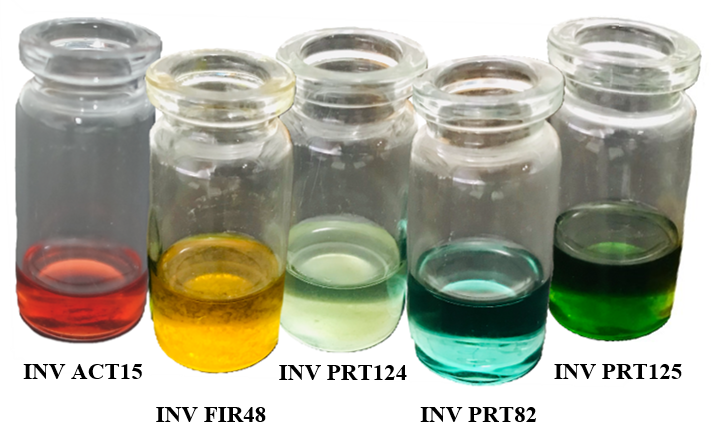


**Figure S4.** Biosurfactants extracted from deep-sea strains Streptomyces sp. INV ACT15, Bacillus sp. INV FIR48, Halomonas sp. INV PRT124, Pseudomonas sp. INV PRT182 and Halomonas sp. INV PRT125


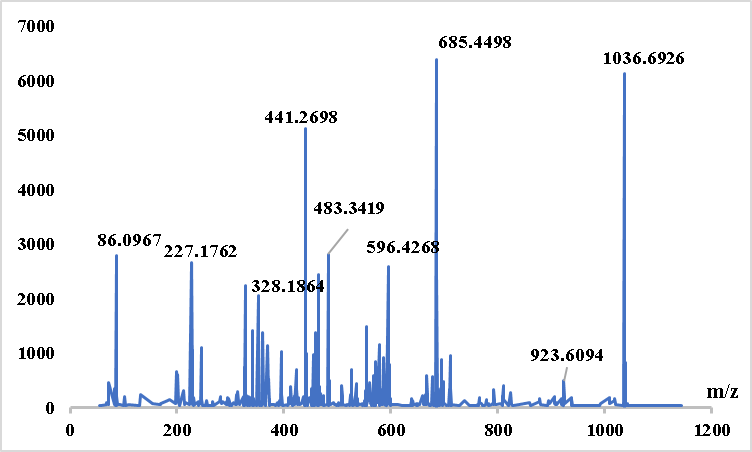


**Figure S5.** Fragmentation spectrum ESI-(+)-MS/MS of ion m/z 1036.6930 identified like esperin in biosurfactants extracts from strains Halomonas sp. INVPRT124, Halomonas sp. INVPRT125, Bacillus sp. INV FIR48 and Pseudomonas sp. INV PRT82.


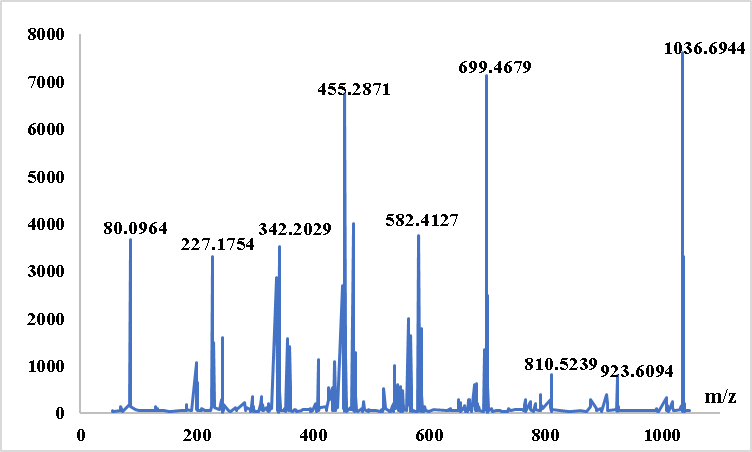


**Figure S6**. Fragmentation spectrum ESI-(+)-MS/MS of ion m/z 1036.6940 identified like [Leu7]surfactin C14i monomethyl ester in biosurfactants extracts from strains Halomonas sp. INVPRT125 and Bacillus sp. INV FIR48


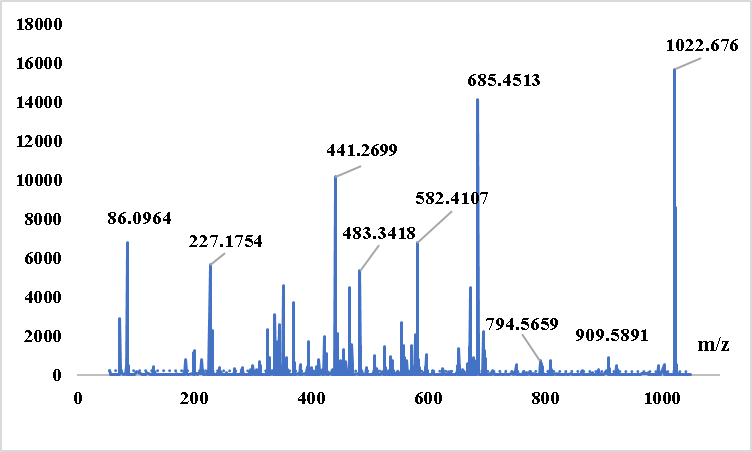


**Figure S7.** Fragmentation spectrum ESI-(+)-MS/MS of ion m/z 1022.6772 identified like Surfactin A C14 in biosurfactants extracts from Halomonas sp. INVPRT124, Halomonas sp. INVPRT125, Bacillus sp. INV FIR48 and Pseudomonas sp. INV PRT82.


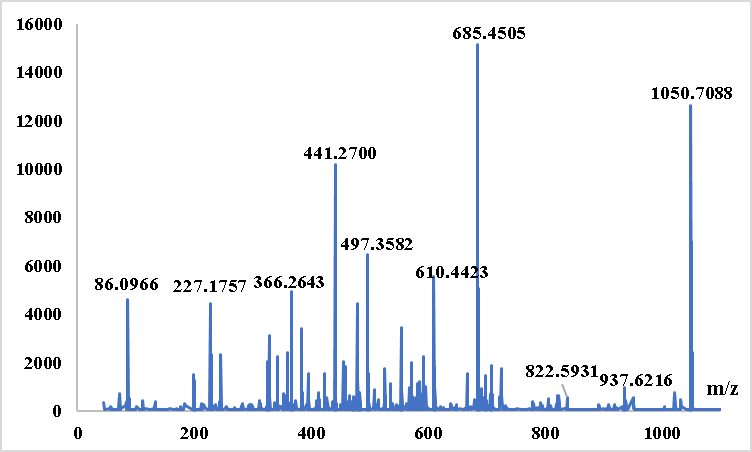


**Figure S8.** Fragmentation spectrum ESI-(+)-MS/MS of ion m/z 1050.7090 identified like Surfactin-D in biosurfactants extracts from strains Halomonas sp. INVPRT124, Halomonas sp. INVPRT125, Bacillus sp. INV FIR48 and Pseudomonas sp. INV PRT82.


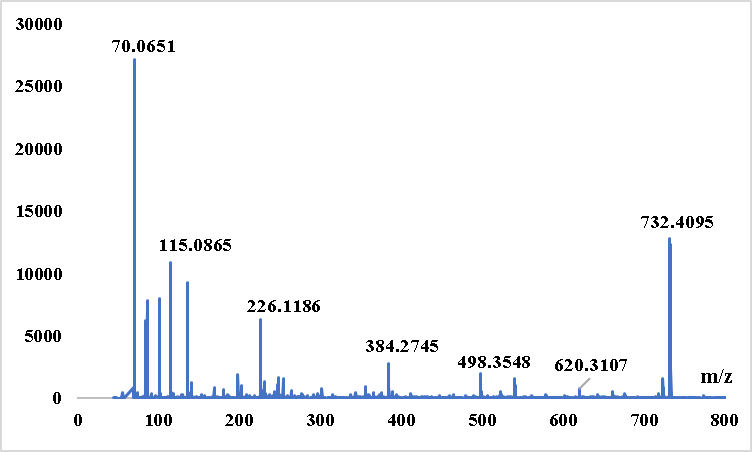


**Figure S9.** Fragmentation spectrum ESI-(+)-MS/MS of ion m/z732,4095 identified like plipastatin in biosurfactants extracts from strains Halomonas sp. INVPRT125 and Pseudomonas sp. INV PRT82.


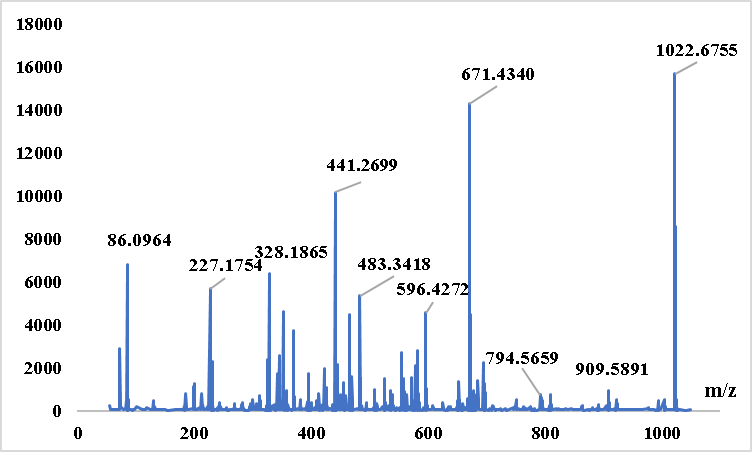


**Figure S10.** Fragmentation spectrum ESI-(+)-MS/MS of ion m/z 1022.6760 identified like [Val7]Surfactin (C15ai) in biosurfactants extracts from strains Halomonas sp. INVPRT124, Halomonas sp. INVPRT125 and Pseudomonas sp. INV PRT82.


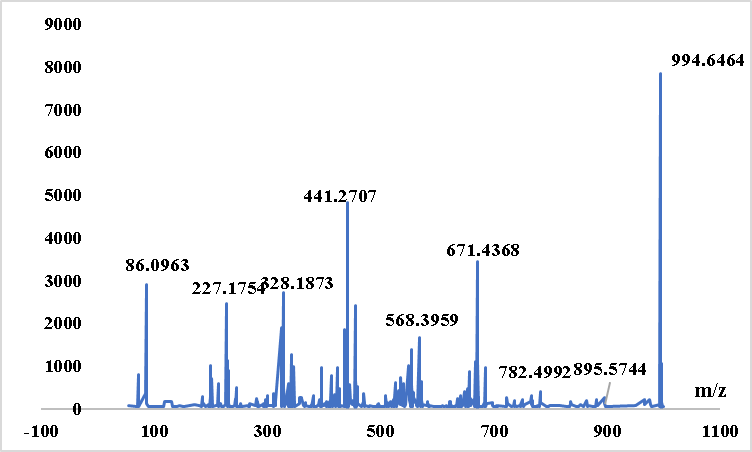


**Figure S11**. Fragmentation spectrum ESI-(+)-MS/MS of ion m/z 994.6452 extracted from molecular network of surfactin family and the fragmentation proposal for isoform [Val7]Surfactin (C13) presents in biosurfactants extracts from Halomonas sp. INVPRT125 and Bacillus sp. INV FIR48.


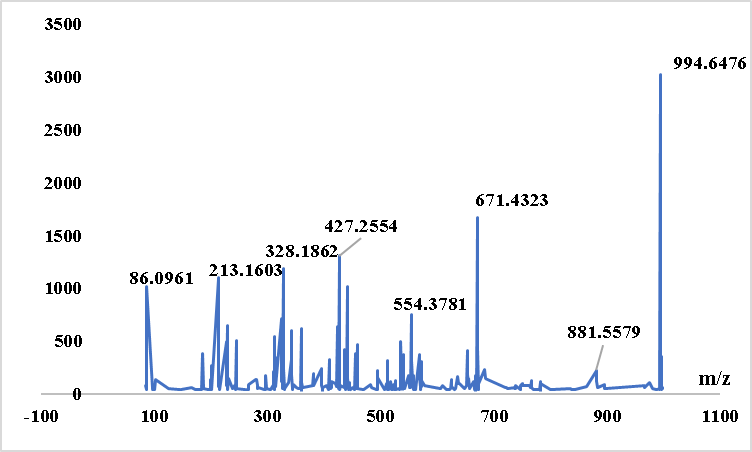


**Figure S12.** Fragmentation spectrum ESI-(+)-MS/MS of ion m/z 994.6449 extracted from molecular network of surfactin family and the fragmentation proposal for isoform Surfactin (C12) presents in biosurfactants extracts from Halomonas sp. INVPRT125 and Bacillus sp. INV FIR48.


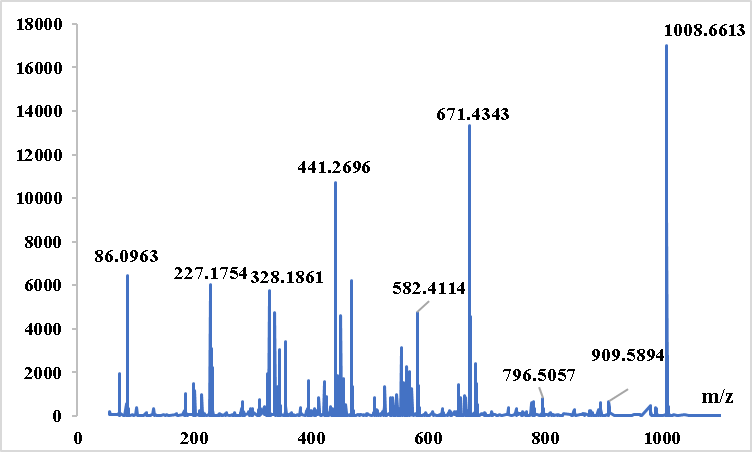


**Figure S13.** Fragmentation spectrum ESI-(+)-MS/MS of ion m/z 1008.6593 extracted from molecular network of surfactin family and the fragmentation proposal for isoform Surfactin A (C13) presents in biosurfactants extracts from Halomonas sp. INVPRT125 and Pseudomonas sp. INV PRT82.


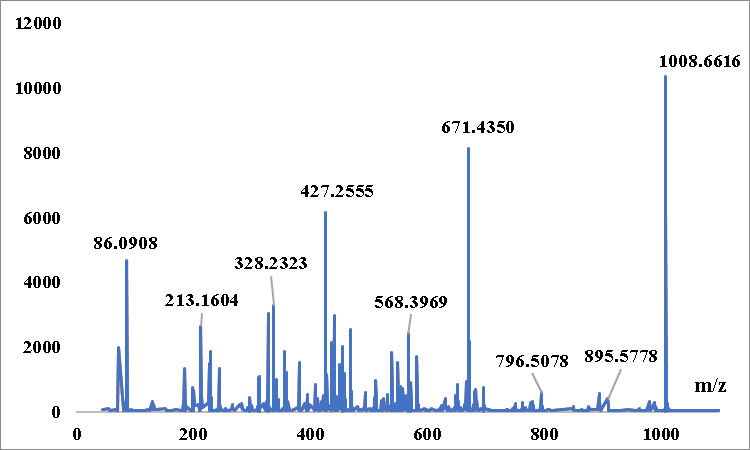


**Figure S14.** Fragmentation spectrum ESI-(+)-MS/MS of ion m/z 1008.6608 extracted from molecular network of surfactin family and the fragmentation proposal for isoform Surfactin (C15) presents in in biosurfactants extracts from Halomonas sp. INVPRT124and Bacillus sp. INV FIR48


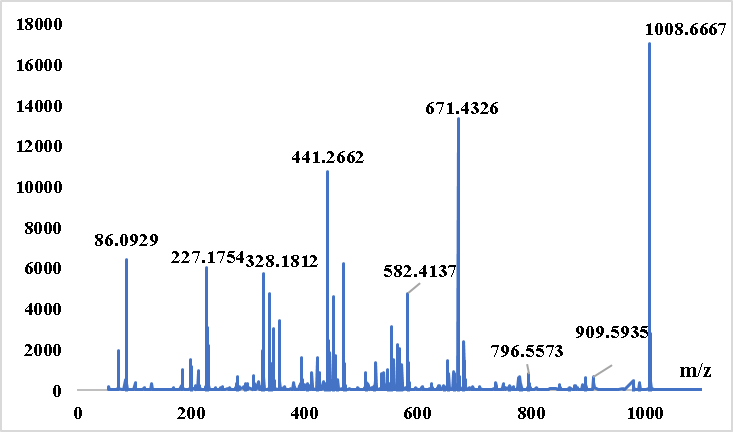


**Figure S15.** Fragmentation spectrum ESI-(+)-MS/MS of ion m/z 1008.6606 extracted from molecular network of surfactin family and the fragmentation proposal for isoform Surfactin A (C13) presents in biosurfactants extracts from Halomonas sp. INVPRT125 and Pseudomonas sp. INV PRT82.


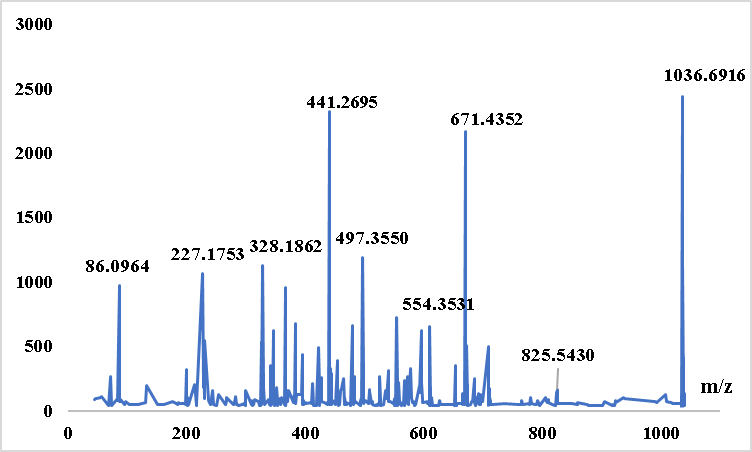


**Figure S16.** Fragmentation spectrum ESI-(+)-MS/MS of ion m/z 1036.6910 extracted from molecular network of surfactin family and the fragmentation proposal for isoform Surfactin B (C16) presents in in biosurfactants extracts from Halomonas sp. INVPRT125 and Pseudomonas sp. INV PRT82.


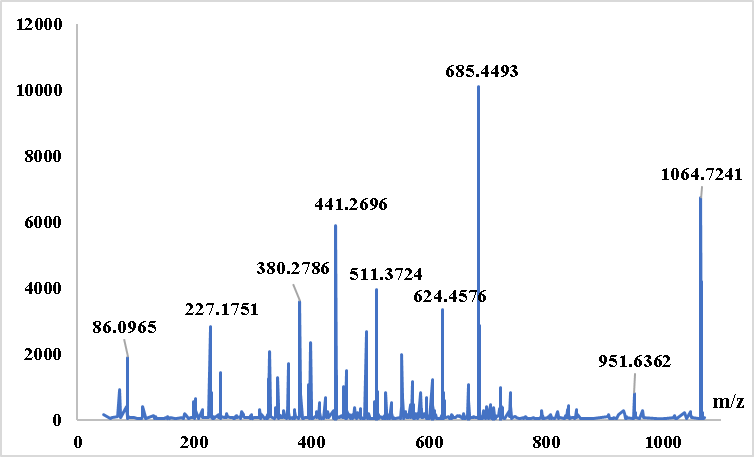


**Figure S17.** Fragmentation spectrum ESI-(+)-MS/MS of ion m/z 1064.7230 extracted from molecular network of surfactin family and the fragmentation proposal for isoform Surfactin A (C17) presents in biosurfactants extracts from Halomonas sp. INVPRT125, Bacillus sp. INV FIR48 and Pseudomonas sp. INV PRT82.


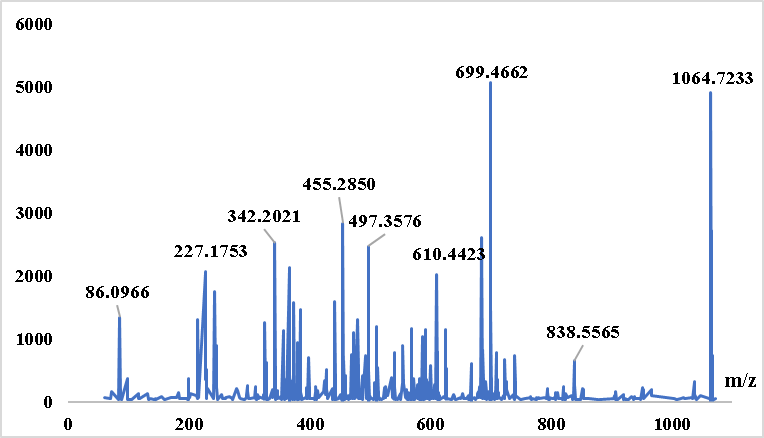


**Figure S18.** Fragmentation spectrum ESI-(+)-MS/MS of ion m/z 1036.6930 extracted from molecular network of surfactin family and the fragmentation proposal for isoform Surfactin monoethyl ester (C16) presents in biosurfactants extracts from Halomonas sp. INVPRT125 and Pseudomonas sp. INV PRT82.

**Table S1.** Center wavenumbers of observed IR peaks in cm−^1^ and their tentative assignment.

The next table has the center wavenumbers of observed IR peaks in cm^−1^ and their tentative assignment. Some of the bands in the fingerprint region are not assigned due to possible ambiguity. str = stretching, bend = bending, sciss = scissoring, wag = wagging, rock = rocking.

| **INV PRT124** | **INVFIR48** | **INVACT15** | **INVPRT125** | **INVPRT82** | **Assignment** |
| --- | --- | --- | --- | --- | --- |
| ν/cm^−1^ | | | | |  |
| 3290 | 3290 | 3300 | 3292 | 3394 | OH str |
| 2920 | 2910 | 2910 | 2918 | 2918 | CH_3_ str |
| 2850 | 2840 | 2850 | 2848 | 2846 | CH_2_ str |
| 1710 | 1720 | 1720 | 1720 | 1728 | C=O str ester |
| 1640 | 1630 | 1640 | 1638 | 1629 | COO str |
| 1200 | 1230 | 1210 | 1275 | - | CH_2_ wag, COH bend |
| 1040 | - | 1040 | 1052 | 1042 | CO str, CH rock |
